# Kissing molars extraction: Case series and review of the literature

**DOI:** 10.4317/jced.52741

**Published:** 2016-02-01

**Authors:** Manuel Arjona-Amo, Eusebio Torres-Carranza, Antonio Batista-Cruzado, Maria-Angeles Serrera-Figallo, Santos Crespo-Torres, Rodolfo Belmonte-Caro, Claudio Albisu-Andrade, Daniel Torres-Lagares, José-Luis Gutiérrez-Pérez

**Affiliations:** 1 Master in Oral Surgery – University Hospital “Virgen del Rocío” Seville, Spain

## Abstract

Kissing molars are a very rare form of inclusion defined as molars included in the same quadrant, with occlusal surfaces contacting each other within a single dental follicle. We present four cases of this pathology: a 35 year-old male, referred to the Oral and Maxillofacial Surgery Department of the Hospital Virgen del Rocio in Seville, and three females of 24, 26, and 31 years, all of which had kissing molars that were treated by tooth extraction. We have found only 10 cases published in the medical literature in which this type of inclusion is briefly described, none of which elaborate on the surgical technique employed. In these cases, the indication for surgery is established when there is a history of recurring infections or cystic lesions associated with dental inclusions. The extraction of kissing molars requires an exhaustive comprehension of the anatomy of the region involved, sufficiently developed surgical abilities, and an extensive planning process.

** Key words:**Impacted molar, kissing molar, surgical extraction.

## Introduction

The term “kissing molar” was first described by Van Hoof in 1973, to refer to molars included in the same quadrant with contacting occlusal surfaces. This phenomenon can occur between the second and third mandibular molars, and cases have also been described of kissing molars between the third inferior and fourth supernumerary molars (1).

Eruption of the second molar occurs on average during the 12th year of age. The second molar erupts between the ages of 20 and 24 years in Caucasians (1).

Development of the third molar tends to commence between the ages of 5 and 14 years, with a maximum intensity of formation during years 8 and 9. X-ray analysis reveals a primary formation bud, in the case of the third lower molar, located near the anterior edge of the mandibular ramus. This bud progressively moves until it is situated distal to the second lower molar. This movement is caused by the migration of the bud within the bone tissue and the changes produced in the mandibular ramus during normal growth. In this manner, the bud initiates in the middle zone of the ramus, above the occlusal plane, but descends vertically and gradually until it is located beneath the occlusal plane. The tendency for mesial migration of the tooth, induced by mastication and normal dental inclinations, facilitates the eruption of the third molar. The reabsorption of the anterior edge of the mandibular ramus also contributes to this. A third mechanism is intrinsic to the formation of the third lower molar. This also requires a change in the inclination of the crown during formation, leaving the bud in a more vertical position. This straightening appears to be influenced by genetic factors (1,2).

Kissing molars are a very rare finding, and we have only found 10 cases described in the medical literature (1-8). However, we were unable to find any articles that described the surgical procedure used to extract these teeth in any detail.

The objective of this article was to present a series of four cases of kissing molars (this being the largest study published on the subject to date), all of which were treated by tooth extraction, with a detailed discussion of the surgical protocol and currently existing literature.

## Case Report

The main characteristics of the clinical cases presented (age, sex, teeth affected, and underlying disease) are summarised in  Table 1. All patients give the informed consent for this use in scientific publications.

**Table 1:**
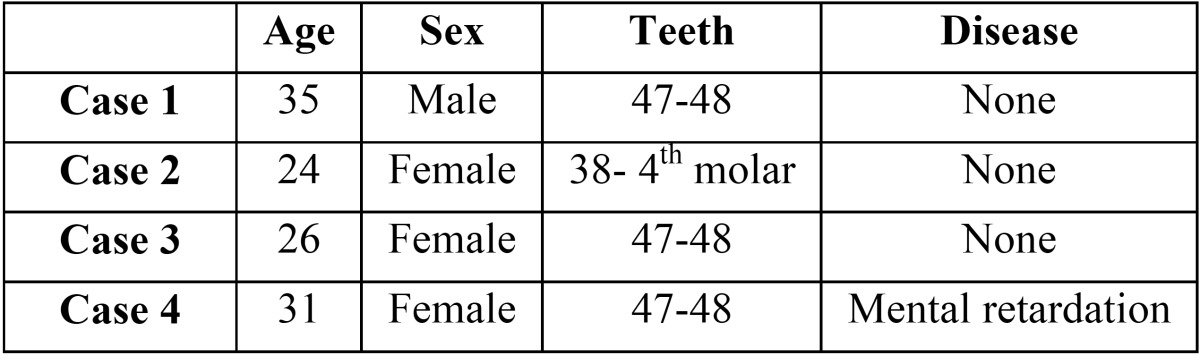
Primary results of the four cases presented.

**Figure 1:**
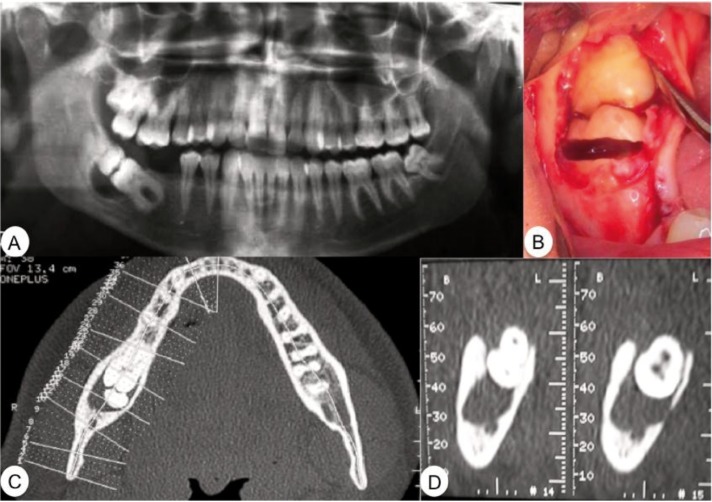
Case 1. A) Preoperative orthopantomogram. B) Cervical tooth section of the crown from tooth 37. C) Axial computed tomography. D) Computed tomography of the relationship between the molars, the dental follicle, and the mandibular canal.

Case 1.- A 35 year-old male sought treatment in the Oral and Maxillofacial Surgery Department of the Hospital Virgen del Rocio in Seville, suffering from pain in the right retromolar region and suppuration with several months’ evolution. The patient did not have any relevant diseases or allergies to medicines, and was currently not taking any medications. We took an orthopantomo-gram, in which the second and third lower right molars were included in the kissing molars position (Fig. 1A).

Given the position of the molars and the follicle and its relationship to the mandibular canal, we also took a computed tomography scan in order to properly plan the surgical treatment (Fig. 1C,D). We performed the surgery under local anaesthesia. This started with a bayonet incision, with a vertical discharge at the distal end of the second premolar, lifting a mucoperiosteal flap.

We then performed an ostectomy using a No. 8 tungsten carbide bur mounted on a hand piece. We performed all necessary tooth sections (separating the crown from the root in both molars) using turbine and fissure burs (Fig. 1B). The large socket created was vigorously cleaned with saline solution and sutured using 4/0 polypropylene monofilament sutures, replacing the tissue flap to its initial position.

Postoperative treatment consisted of amoxicillin/clavulanic acid at 875mg/125mg every 8 hours for five days, and ibuprofen at 600mg every 8 hours for five days. The sutures were removed after 8 days. Ten days after the procedure, there were no signs of infection or anaesthesia/paraesthesia in the inferior alveolar nerve.

Case 2.- Female patient of 24 years of age was referred to the Oral and Maxillofacial Surgery Department of the Hospital Virgen del Rocio in Seville by an orthodontist due to two included molars with contacting occlusal surfaces and discomfort in the area with several months’ evolution.

**Figure 2:**
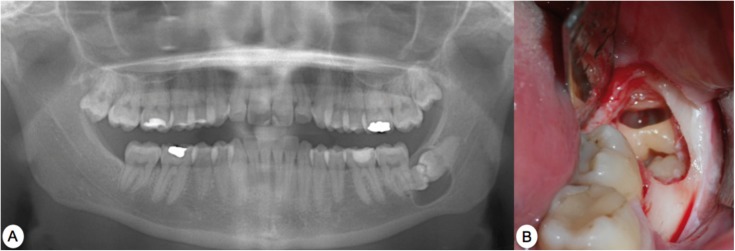
Case 2. A) Preoperative orthopantomogram. B) Vestibulo-lingual tooth section of the supernumerary molar crown.

An orthopantomogram revealed kissing molars between tooth 38 and the fourth supernumerary molar, as well as a large radiolu-cent area surrounding the crowns of both teeth (Fig. 2A).

In addition to the position of the molars and the orthodontist’s indications, the suspicion of a follicular cyst provided the indication for extraction of both molars and a pathological analysis of the lesion around the crowns.

We surgically removed the kissing molars under local anaesthesia. We performed the incision, tissue flap, and ostectomy using hand tools and a No. 8 surgical bur until reaching the crowns of the two teeth. We then performed a cervical tooth section in order to extract the crowns and the follicle along with the cyst that surrounded them (Fig. 2B). We then extracted the roots separately and finished with 4/0 silk sutures.

We administered antibiotic and anti-inflammatory treatment under a similar prescription to the first case. We also administered intramuscular methylprednisolone (Urbason® at 80mg) in order to reduce postoperative oedema and inflammation. The patient had satisfactory evolution and developed no postoperative complications. The histopathological analysis confirmed the presence of a follicular cyst.

Case 3.- A female patient of 26 years of age sought treatment at the Oral and Maxillofacial Surgery Department of the Hospital Virgen del Rocio in Seville, with right hemifacial pain of six months evolution. An intraoral examination revealed poor oral hygiene and root remnants with several missing teeth.

**Figure 3:**
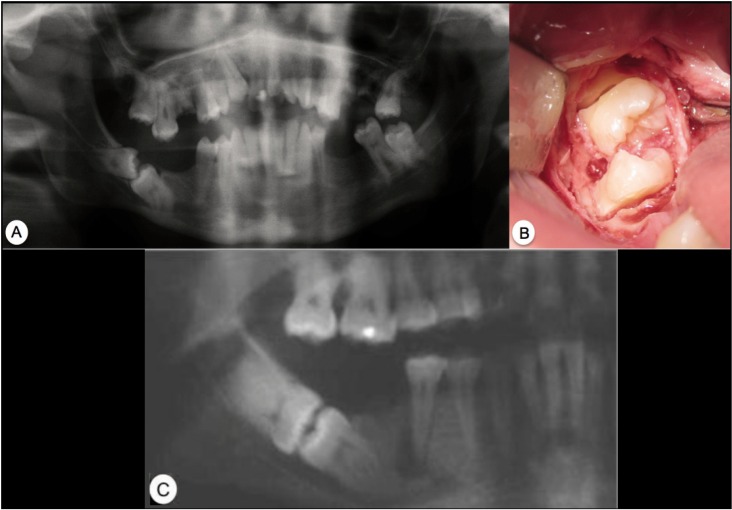
Case 3. A) Preoperative orthopantomogram. B) Observe the tissue flap, the ostectomy and tooth sections, separating the crown from the roots, just before being removed using forceps. C) Case 4. Preoperative orthopantomogram.

Palpations revealed severe pain and inflammation in the area of the right lower molars. An orthopantomogram revealed two kissing molars (47 and 48) (Fig. 3A).

We established a treatment plan involving tooth extraction under local anaesthesia. We performed a bayonet incision, extending it distally so as to gain adequate access to the kissing molars. We then performed an ostectomy, eliminating the bone covering the crowns, yielding access for performing the tooth sections. In this case, we performed the tooth section of the crowns from both molars at the same time (Fig. 3B), and then proceeded to extract each one separately. Once the crowns were extracted, we removed first the lower molar root and then the upper molar root.

In this case, the removal of the two kissing molars left a large defect in the bone and severe mandibular weakening, prompting the placement of a cow bone graft in order to accelerate bone regeneration. We finally sutured the wound using 4/0 silk sutures.

Following the procedure, we administered intramuscular methylprednisolone (Urbason® at 80mg) and started the patient on a similar antibiotic and anti-inflammatory treatment regimen to that of cases 1 and 2.

Case 4.- A female patient of 31 years of age with slight mental retardation sought treatment at the Oral and Maxillofacial Surgery Department of the Hospital Virgen del Rocio in Seville, complaining of right hemifacial pain and inflammation with several months’ evolution. The orthopantomogram revealed kissing molars between teeth 47 and 48 (Fig. 3C).

We removed the kissing molars using the same technique described in previous cases. We provided anaesthetic blocks of the inferior alveolar, lingual, and buccal nerves. We then followed with a bayonet incision and lifted the tissue flap, proceeding with the ostectomy using hand tools and a No.8 tungsten carbide bur with copious irrigation in order to expose the two crowns and part of the root surface. After the ostectomy, we performed the cervical tooth sections using turbine and fissure burs in order to first extract the crown and then the root fragment. We first removed the upper molar and then the lower. The tissue flap was sutured using 4/0 silk sutures, and the patient was administered intramuscular methylprednisolone (Urbason® at 80mg) and the same antibiotic and anti-inflammatory treatments as before.

## Discussion

In 1973, Van Hoof (1) first used the term “kissing molars” to describe the clinical case of a patient with mental retardation who had this type of inclusion bilaterally ( Table 2). Several years later, in 1991, Robinson and colleagues (2) presented the case of a young woman with bilateral kissing molars between the second and third mandibular molars. In this case, the medical history of the patient contained no abnormalities.

**Table 2:**
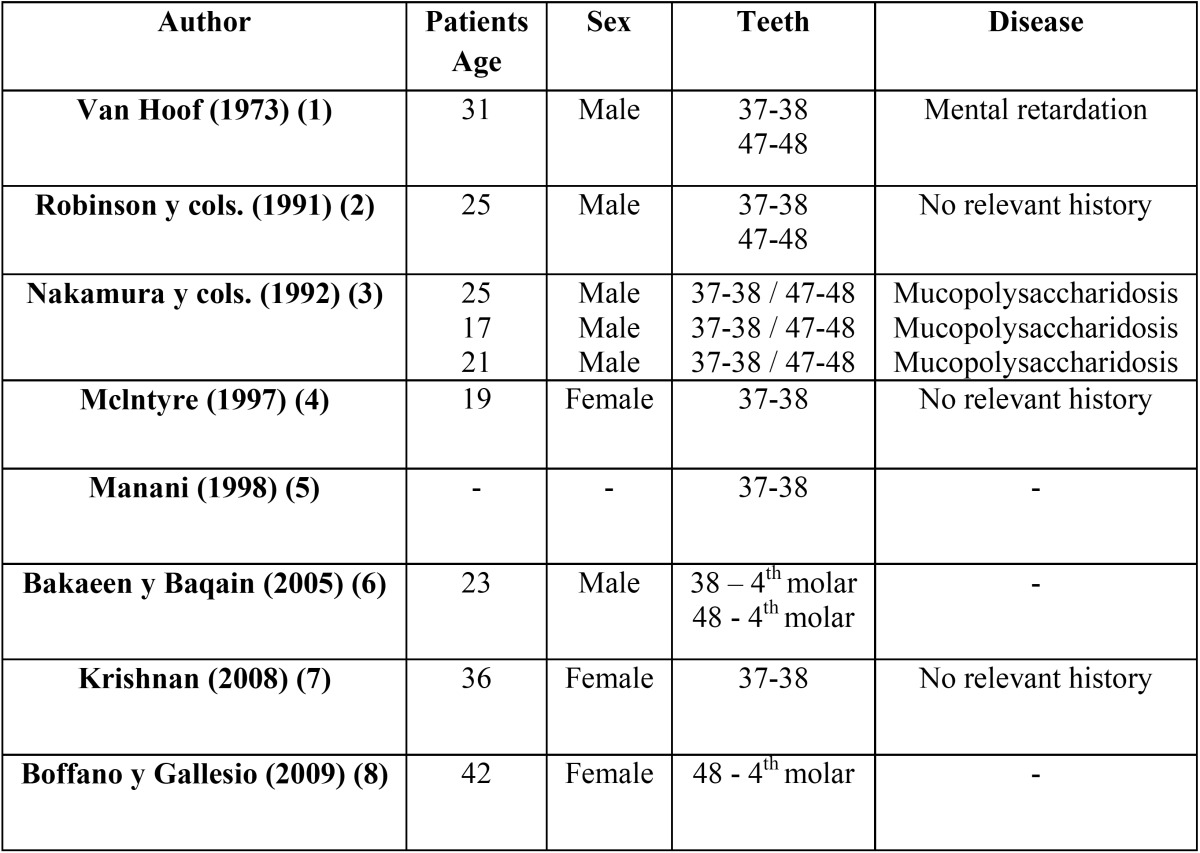
Main publications regarding kissing molars in international journals (MedLine) and the English language (Key words for the search: “kissing molar” – Limits: English – Database: MedLine; we eliminated the article by Juneja (9) since it was a commentary, and added the article by Nakamura and colleagues (3), which was obtained by reviewing the citations from the articles obtained in the initial literature search).

Nakamura and colleagues (3) presented a series of four cases of patients diagnosed with mucopolysaccharidosis in which, in addition to other disorders, three patients had multiple kissing molars. These molars were contained within single dental follicles. Nakamura concluded that this pathology was suggestive of mucopolysaccharidosis, although it can also occur independently.

In 1997, MchIntre (4) presented the case of unilateral kissing molars of the second and third lower left molars. In these observations, the lower dental canal was apically displaced due to the migration of the second molar. Manani (5) presented a very similar case, and introduced the term of inverted impaction to describe this phenomenon. In 2005, Bakaeen and Baqain (6) published one case of bilateral kissing molars between the third mandibular and fourth supernumerary molars.

In 2008, Krishnan (7) presented the case of a patient in which the second and third left lower molars shared a single follicle, based on his opinion. The pathological analysis of the patient’s follicle showed this to be a dentigerous cyst. Krishnan explained this finding as follows: possible bone reabsorption by the expansion of a dentigerous cyst associated with a second included molar caused the loss of the bone wall of the mesial root of the third molar, causing the occlusal surface of the third molar to come into contact with the second molar as the structure became inclined.

In 2009, Boffano and Gallesio (8) published the last case described until now, in which they observed the inclusion of the third mandibular molar and fourth supernumerary molar characteristic of kissing molars.

According to Krishnan (7), the important characteristic of kissing molars is that they share a single follicle and have contacting occlusal surfaces. In accordance with Juneja (9), we believe that if there is a dentigerous cyst in any of the teeth involved, they could not share the same follicle as described in the case by Krishnan. There would also be no contact between the impacted molars. As Juneja opined, this would be an unusual case of impaction rather than one of kissing molars.

Nakamura and colleagues (3) have associated kissing molars with mucopolysaccharidosis. As Smith and colleagues (10) argued, this could be due to the delayed eruption of permanent teeth in patients with mucopolysaccharidosis. If early surgical exposure of the teeth is not carried out, before the teeth move to the lower rim of the mandibular ramus, the possibility of eruption will be minimal since the follicle expands such that it impedes eruption and the teeth remain included. Impacted teeth would thus need to be extracted.

We cannot at this point rule out this relationship, but as we can observe in the scant literature currently available on the subject, kissing molars can appear in patients with no underlying systemic pathologies, and so this possible relationship should be explored in depth.

## Conclusions

Dental inclusion of kissing molars constitutes a rare clinical entity. Indications for surgery involve a history of recurring infections or cystic lesions associated with the dental inclusions. The surgical approach for this condition requires an exhaustive understanding of the anatomy of the region, advanced surgical abilities, and a rigorous planning process. Little scientific knowledge has been gained in relation to this pathology, and a greater number of publications are needed on this topic.
